# Do Gender-Specific and High-Resolution Three Dimensional Body Charts Facilitate the Communication of Pain for Women? A Quantitative and Qualitative Study

**DOI:** 10.2196/humanfactors.5693

**Published:** 2016-07-20

**Authors:** Line Lindhardt Egsgaard, Trine Søby Christensen, Ida Munk Petersen, Dorthe Scavenius Brønnum, Shellie Ann Boudreau

**Affiliations:** ^1^Faculty of MedicineSMI®, Department of Health Science and TechnologyAalborg UniversityAalborg ØstDenmark; ^2^Faculty of MedicineDepartment of Health Science and Technology, School of MedicineAalborg UniversityAalborg ØstDenmark

**Keywords:** mHealth, app, android, pain measurement, chronic pain, three dimensional pain drawing, digital communication

## Abstract

**Background:**

Chronic pain is more prevalent among women; however, the majority of standardized pain drawings are often collected using male-like androgynous body representations.

**Objective:**

The purpose of this study was to assess whether gender-specific and high-resolution three-dimensional (3D) body charts facilitate the communication of pain for women.

**Methods:**

Using mixed-methods and a cross-over design, female patients with chronic pain were asked to provide detailed drawings of their current pain on masculine and feminine two-dimensional (2D) body schemas (N=41, Part I) or on female 2D and 3D high-resolution body schemas (N=41, Part II) on a computer tablet. The consistency of the drawings between body charts were assessed by intraclass correlation coefficient (ICC) and Bland-Altman plots. Semistructured interviews and a preference questionnaire were then used to obtain qualitative and quantitative responses of the drawing experience.

**Results:**

The consistency between body charts were high (Part I: ICC=0.980, Part II: ICC=0.994). The preference ratio for the masculine to feminine body schemas were 6:35 and 18:23 for the 2D to 3D female body charts. Patients reported that the 3D body chart enabled a more accurate expression of their pain due to the detailed contours of the musculature and bone structure, however, patients also reported the 3D body chart was too human and believed that skin-like appearance limited ‘deep pain’ expressions.

**Conclusions:**

Providing gender-specific body charts may facilitate the communication of pain and the level of detail (2D vs 3D body charts) should be used according to patients’ needs.

## Introduction

Pain is the primary symptom for 40% of all visits to the primary care physician [1]. The most common cause of pain is of musculoskeletal origin [1,2], and almost all anatomical sites are reported to have a higher prevalence of chronic pain for women [3]. Additionally, the prevalence for neuropathic, widespread, and abdominal pain is also higher among women [4]. Various tools and questionnaires have been developed to document and communicate a patient’s pain experience and associated symptoms to health care professionals and researchers [5]. Of these methods, pain drawings are widely used to communicate pain extent and location as the drawings can depict symptoms and ultimately assist in diagnosis [6-9]. Traditionally, paper versions of two-dimensional (2D) outlines of the body are provided to the patient for them to indicate and draw the area or pattern of their perceived pain. These traditional 2D outlines of the body are deemed androgynous but they are clearly more masculine [8-11] and whether this influences a woman’s ability to clearly express the extent and location of her pain is unknown. Androgynous body charts can hide clinically relevant anatomical differences between the genders, such as the width and contour of the hips, waist, chest, and shoulders.

An accurate and careful assessment and communication of pain from patient to a health care professional is an essential step toward diagnosis and pain management [12]. However, assessment and communication of pain are influenced by two types of error: the assessor and the communication tool. When using 2D androgynous body charts women with chronic musculoskeletal pain report similar pain intensities to their male counterparts; however, women tend to report slightly larger pain areas than men in all anatomical sites [13]. Is this difference in pain area between the genders a true depiction and is there any clinical relevance between the differences? Few studies, have employed gender-specific or more feminine body charts [14-16]; however, no studies have cross-validated a female to a male body chart nor investigated whether the patient prefers using gender-specific body charts for expressing and communicating their pain. Indeed, it has been proposed that men and women experience and communicate pain differently [17], the question is whether a female body chart provides the otherwise missing and necessary anatomical guidance required for women to more clearly and accurately express their pain; and if so, does the use of high-resolution, three-dimensional (3D) body charts further improve this form of communication?

The aim of this study was to determine whether a feminine, as compared with a masculine version, of a 2D body chart is preferred by women for the communication of pain extent and to evaluate drawing behavior by assessing the level of agreement between the drawn pain areas between the gender body charts. In a similar fashion, this study set out to determine whether enhanced anatomical detail would further improve the ability to express current pain. It was hypothesized that female patients would prefer a feminine body chart with enhanced anatomical detail and that drawing behavior would be influenced by the gender of the body chart.

## Methods

### Overview

**Figure 1:**
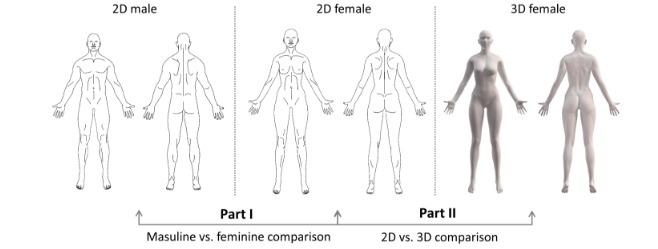
Overview of the body charts compared in Part I and II. Part I compares the masculine and feminine body charts and Part II compares the female 2D and 3D body charts.

This mixed-methods study was conducted with female patients referred to a multidisciplinary pain clinic for the purpose of chronic pain management, in order to assess the drawing behavior, preference, perception, and drawing experience of using masculine and feminine body charts (Part I) or traditional 2D line and high-resolution 3D female body charts (Part II), as shown in Figure 1. A randomized cross-over design for both Part I and II was implemented. All participants were asked to indicate the area and location of their current pain on two body charts in randomized order, in accord with either Part I or II. A questionnaire was administered to assess preference of body chart immediately after the pain drawings. Further, a semistructured interview was conducted in order to assess the user experience and the impact of using body charts with (Part I) masculine and feminine features or (Part II) enhanced anatomical detail for the communication of pain extent.

In Denmark, approval from the local ethics committee for survey and interview studies is not legally required. Nevertheless, this study was performed in adherence to ethical rules and guidelines with respect to voluntary participation and confidentiality and the study was reported to the Danish data protection agency. Signed informed consent was obtained before participation and the study was conducted according to the Helsinki Declaration.

### Recruitment

**Figure 2:**
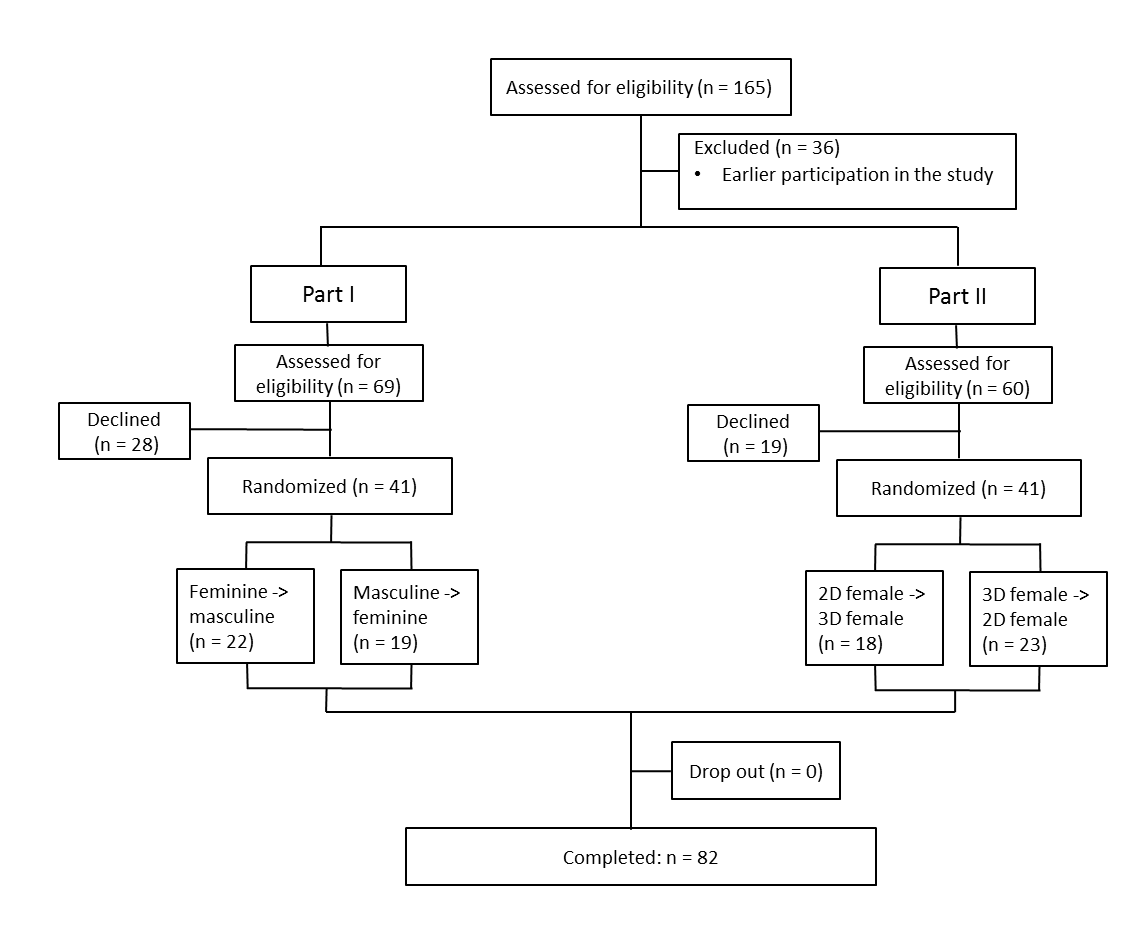
CONSORT flow diagram. The progress of participants through the study is shown. Group Part I=comparison of masculine and feminine body charts; group Part II = comparison of female 2D and 3D body charts.

Female patients were recruited from the waiting room of a multidisciplinary pain clinic (Tværfagligt Smertecenter, Aalborg, Denmark), and therefore represent a convenience sample consisting of heterogeneous diagnosis (Table 1). Individuals with chronic pain and corrective vision were included, and those with known neurologic or movement disorders that could potentially affect motor control of the hand-eye coordination or drawing ability were excluded. For this study a total of 82 patients agreed to participate, resulting in 41 participants (mean age: 43.3±15.9, range: 18-84) in Part I and 41 participants (mean age: 40.6±14.2, range: 20-69) in Part II (see Figure 2 for CONSORT diagram).

**Table 1:** The distribution of patient's self-reported diagnosis divided into categories of musculoskeletal, neuropathic, visceral, and idiopathic pain; diagnosis not fitting in the four main categories are placed in the “other” category.

Category	Part I	Part II
	n (%)	n (%)
Musculoskeletal pain	18 (44)	23 (56)
Neuropathic pain	4 (10)	7 (17)
Visceral pain	2 (5)	0 (0)
Idiopathic pain	10 (24)	8 (19)
Other	7 (17)	3 (7)

### Body Charts, Tablet, and Drawing Pen

Drawings of pain extent and location were collected on a Samsung Galaxy Note 10.1 tablet with Android 4.1.2 (Jelly Bean) using the Navigate Pain app. A digital display of the masculine, feminine, 2D, and 3D body charts were viewable on the tablet screen. Participants were asked to draw with an S Pen (pen tip is approximately 1.5 mm), which is an accessory that accompanies the Samsung Galaxy Note 10.1 tablet. In order to make the recording conditions as similar as possible, the thickness of the line created by the S Pen was kept the same (~10 pixels). Participants were asked to draw the area(s) of their current pain as accurately as possible and to the best of their ability. The masculine and feminine 2D line drawings depicted main landmark features, such as the knee, elbows, and navel, whereas the 3D female body chart depicted both main landmark features and contour shadings. A short time-interval (˂1 minute) between the administrations of each body chart version was chosen to minimize variation in pain extent and location between the two body chart versions.

### Assessment of Preferences and Perception of Body Charts

Immediately following completion of the pain drawings, participants were asked to fill out a short questionnaire in order to determine the preference and drawing experience between the masculine and feminine or 2D and 3D body charts. The evaluation questionnaire consisted of three questions, one assessing preference of body chart (two alternative forced choices), and two questions assessing the drawing experience of the two body charts on a 7-point Likert scale (very difficult, difficult, slightly difficult, neutral, slightly easy, easy, very easy).

### Interviews With the Participants

Both Parts I and II of the study concluded with a semistructured interview (~10 minutes). The semistructured interview consisted of open-ended questions where participants could explain and provide the reasons for their choices. The purpose of the interview was to gain insight and a detailed understanding of the participants’ preferences and drawing/user experience, specifically: perception of body chart and drawing experience, identification with the body chart, and suggested improvements.

During the semistructured interviews, thorough notes were taken including precise quotes from each participant. The qualitative data from the interviews was analyzed using thematic content analysis inspired by Kvale [18]. On the basis of 41 participants in each of the two parts of the study, it was possible to deduce and isolate typical and unique themes/characteristics.

### Digital Quantification of Pain Area

The pain areas marked on the body charts were objectively quantified using the Navigate Pain software as total number of pixels and expressed as a percentage of the total drawable pixels in each view of the body chart (pixel density). The 2D female body chart drawing is an outline of the 3D female body chart and the total drawable pixels for the 2D female body chart are 194,542 pixels on the anterior view and 200,309 pixels on the posterior view, and 188,611 pixels on the anterior view and 194,096 pixels on the posterior view for the 3D female body chart. The masculine body chart has 194,922 pixels on the anterior view and 204,410 pixels on the posterior view. For participants requiring two views of the body charts, that is an anterior and posterior perspective, to express their pain area(s), the average pixel density of the two views was used for statistical analysis so that the total number of drawable pixels was equivalent between subjects and comparisons.

### Statistical Analysis

In order to determine the consistency of the drawn pain areas between the different body charts a reliability analysis on pixel density was performed by computing a two-way, mixed-model (test value=0) intraclass correlation coefficient (ICC) between the masculine versus feminine and between the 2D versus 3D female body charts. In order to determine if the natural variation in the drawing behavior was maintained across methods, a Levene’s test for homogeneity (one-way analysis of the variance) was used on the pixel density to test for equal variance within masculine versus feminine and 2D versus 3D body charts. Further, a one-sample *t* test comparing the difference in pixel density (subtracting masculine from feminine; 2D from 3D) to zero was performed to test for differences in the size of the drawings between the body charts. In order to understand any differences in drawing size between the body charts a Bland-Altman plot with 95% limits of agreement (LOA) was used to investigate the level of agreement in pixel density between masculine versus feminine and 2D versus 3D body charts. A systematic disagreement in pixel density between masculine versus feminine and 2D versus 3D body charts was defined as a fixed bias (a difference in drawn area between the body charts is a constant) and proportional bias (a difference in the drawn area between the body charts is a factor). Fixed bias was assessed by using the calculated mean of the difference in pixel density from the Bland-Altman plot. Proportional bias was assessed by a two-tailed Pearson correlation between the difference in pixel density and the mean in pixel density from the Bland-Altman plot. Absolute proportional bias was assessed by a two-tailed Pearson correlation on the rectified data from the Bland-Altman plot (absolute error, rectified difference between the drawn areas on the body charts is a factor). All statistics were performed in SPSS 22 and α=0.05 was used as level of significance. Results are presented in mean ± standard deviation (SD).

## Results

### Comparison of Drawn Pain Area Between the Masculine and Feminine Body Charts (Part I)

**Figure 3:**
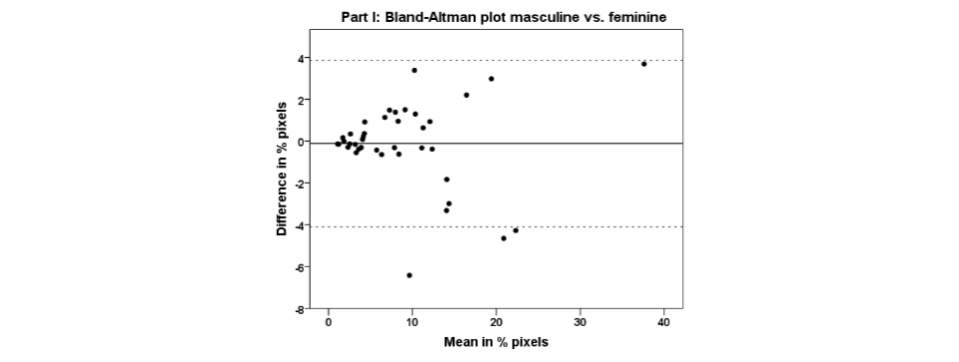
Bland-Altman plot for Part I: masculine compared with feminine body charts. The data is presented in % pixels. The data on the x-axis is the mean of the pain areas drawn in the two body charts and the data on the y-axis is the difference between the pain areas drawn in the two body charts. The dashed lines illustrate the 95% LOA.

Two outliers were excluded because the difference between the pixel densities of the masculine and feminine body charts were more than 2 SD away from the group mean. The LOA between the pixel densities of masculine and feminine body charts was high (ICC=0.98, F=51.15, df=38, *P*<.001). One-sample *t* test of the difference between the pixel densities of the masculine and feminine body charts was not significant (mean difference=−0.12 ± 2.04; *t*=−0.365, *P*=.717). Levene’s test for homogeneity showed no statistical difference in variance between the pixel densities of the masculine and feminine body charts (Levene statistic=0.038, *P*=.943). A Bland-Altman plot for comparing the pixel densities of the masculine and feminine body charts (Figure 3) shows a mean difference in pixel density of −0.12%; with upper and lower LOA 3.87% and −4.11%, respectively. A fixed-negative bias was found (−0.12) indicating that the pain areas were drawn slightly smaller on the masculine than on the feminine body chart. No proportional bias was found between the pixel densities of the masculine and feminine body charts (Pearson correlation=−0.002, *P*=.991). Most notably, an absolute proportional bias was found between the pixel densities of the masculine and the feminine body charts (Pearson correlation=0.694, *P*<.001) indicating that the difference in drawn areas became larger when patients report larger areas of pain.

### Comparison of Drawn Pain Area Between the Two- and Three-Dimensional Female Body Charts (Part II)

**Figure 4:**
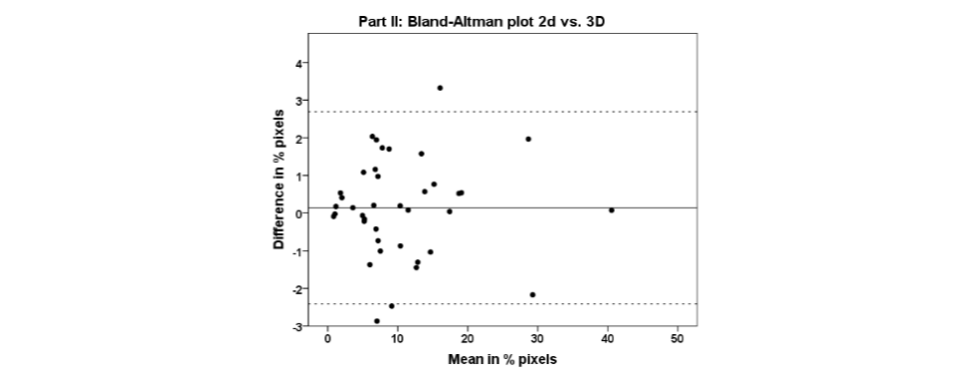
Bland-Altman plot for Part II: female 2D and 3D body charts. The data is presented in % pixels. The data on the x-axis is the mean of the pain areas drawn in the two body charts and the data on the y-axis is the difference between the pain areas drawn in the two body charts. The grey dashed lines illustrate the 95% LOA.

Two outliers were excluded because the difference between the pixel densities of the 2D and 3D female body charts were more than 2 SD away from the group mean. The LOA between the pixel densities of 2D and 3D female body charts was high (ICC=0.994, F=161.888, df=38, *P*<.001). One-sample *t* test of the difference between the pixel densities of the 2D and 3D female body charts was not significant (mean difference=0.14±1.30; *t*=0.674, *P*=.504). Levene’s test for homogeneity showed no statistical difference in variance between the pixel densities of the 2D and 3D female body charts (Levene statistic=0.002, *P*=.963). A Bland-Altman plot for comparing the pixel densities of the 2D and 3D female body charts (Figure 4) shows a mean difference of 0.14%; with upper and lower LOA of 2.69 and −2.41, respectively. A fixed-negative bias was found (0.14) indicating that pain areas were drawn marginally larger on the 3D than on the 2D female body chart. No proportional bias was found between the pixel densities of the 2D and 3D female body charts (Pearson correlation=0.016, *P*=.924). Unlike the comparison between the pixel densities of the masculine and feminine body charts, no absolute proportional bias was found between the pixel densities of the 2D and 3D female body charts (Pearson correlation=0.181, *P*=.271).

### Preference of Body Charts and Drawing Experience

Preference was assessed by two alternative forced choices. The level of difficulty for drawing and expressing pain extent and location was assessed by a 7-point Likert scale where 4 was ‘neutral’ and 3 levels of difficult/easy could be chosen on each side of neutral. For simplicity the results are pooled on each side of ‘neutral’.

#### Masculine and Feminine Two-Dimensional Body Charts (Part I)

The distribution of masculine and feminine body chart preferences are outlined in Table 2. With respect to the drawing experience on the feminine body chart, only 1 participant reported some degree of difficulty, 1 participant was neutral, and remaining 39 participants reported some degree of easiness in drawing their pain. The participant who reported difficulty in expressing pain on the feminine body chart also indicated a preference for the masculine body chart. With respect to the drawing experience on the masculine body chart, only 5 participants reported some degree of difficulty, two participants were neutral, and 34 participants reported some degree of easiness in drawing their pain. Notably, 4 of the participants who reported difficulty in expressing pain on the masculine body chart also indicated a preference for the feminine body chart.

**Table 2:** the distribution of body chart preferences in response to the forced choice questionnaire and subsequent qualitative assessment.

		Body chart preference	
		Quantitative – Forced choice	Qualitative assessment
		n (%)	n (%)
**Part I (N=41)**			
	Feminine	35 (85)	18 (44)
	Masculine	6 (15)	2 (5)
	Ambivalent	0 (0)	21 (51)
**Part II (N=41)**			
	Two-dimensional female	18 (44)	16 (39)
	Three-dimensional female	23 (56)	20 (49)
	Ambivalent	0 (0)	5 (12)

#### Two- and Three-Dimensional Female Body Charts (Part II)

The distribution of 2D and 3D female body chart preferences are outlined in Table 2. With respect to the drawing experience on the 2D female body chart, only 5 participants reported some degree of difficulty, 1 participant was neutral, and 35 participants reported some degree of easiness in drawing their pain. The 5 participants who reported difficulty in expressing their pain on the 2D female body chart clearly preferred the 3D female body chart. On the other hand, with respect to the drawing experience on the 3D female body chart, 5 participants reported some degree of difficulty, 1 participant was neutral, and 35 participants reported some degree of easiness in drawing their pain. Two participants who reported difficulty in expressing pain on the 3D female body chart preferred the 2D female body chart; however, 1 participant still preferred the 3D female body chart. The 2 remaining participants reported difficulty in expressing pain on both the 2D and 3D body charts.

#### Semistructured Interviews

Five identical themes were identified for the semistructured interviews in Part I and II: difference between body charts, preference (explained), identification with the body charts, accuracy/drawing experience, and improvements. The themes and associated quotes are compiled in Tables 3 and 4 for Part I and II, respectively. The quotes presented in Tables 3 and 4 are translated from Danish to English with emphasis on the meaning of the content and not the direct translation.

#### Masculine Version Feminine Two-Dimensional Body Charts (Part I)

The semistructured interviews investigating preference and drawing experience between the masculine and feminine 2D body charts (Table 3) revealed that the 2 presented body charts were indeed, perceived as feminine and masculine. However, for some participants the gender difference between the body charts was not apparent until both body charts were presented simultaneously. When explaining the preference for a specific body chart, half of the participants indicated that the choice was random or that it did not matter which body chart they used (21/41, 51%). However, for a number of participants (18/41, 44%) it was very important that the body chart was female and the preference for the female body chart felt natural. Interestingly, only 2 participants truly preferred the masculine body chart and this was attributed to the familiarity of this body chart and the feeling of anonymity when expressing their pain. When asked about identification with the body charts, some participants expressed that identification itself was not important or that they could identify with both body charts. For some participants identification with the (feminine) body chart was very important because it enabled a more accurate and personal expression of their pain. Only 1 participant identified more with the masculine body chart and this was attributed to the perception that this body chart was larger than the feminine body chart (whole body); despite the fact the actual true difference in size is 0.4%. Regarding the drawing experience, participants mainly focused on the ability to reproduce their pain pattern. For the feminine body chart, the curves and shapes (hips, waist, breasts, and shoulders) were emphasized as being important factors for an accurate portrayal or communication of pain. Some participants found ‘it took more thought’ to project their pain pattern onto a masculine body chart. However, for 1 participant, who preferred and identified with the masculine body chart, the perception of a larger representation of specific areas on the masculine body chart was important for an accurate communication of pain. The suggestions for improvements when communicating their pain on the body charts were the option to indicate the quality and intensity their pain and clear indications of left and right body sides as this could lead to confusion when drawing different areas of pain.

### Two- Versus Three-Dimensional Female Body Charts (Part II)

The semistructured interviews investigating the preference and drawing experience between the 2D and 3D female body charts (Table 4) indicated that the 2D female body chart was perceived as more ‘anonymous’, ‘clinical’, and ‘natural’; in comparison to the 3D female body chart, which was perceived as ‘human’, ‘alive’, and ‘more detailed’. However, the 2D female body chart was also perceived as ‘artificial’ or ‘flat’ and the 3D female body chart as ‘alien-like’ or ‘robotic’. When explaining the preference for a specific body chart, the clear dichotomy in preference between the 2D and 3D body charts appears to be influenced by implicit attitudes and perception of detail in the specific body chart. When asked about identification with the body charts, a few participants expressed that they did not identify with any of the body charts or that identification with the body charts was unimportant. However, the majority of participants identified with the body charts and indicated that identification was an important factor for expressing pain accurately on a body chart. Similar to the participants who compared feminine and masculine 2D body charts, the drawing experience was expressed as the ability to reproduce the pain pattern accurately (or not being able to). Those participants who indicated that their pain was best reproduced or accurately communicated on the 2D female body chart, the level of detail, the lines, and the fact that it was ‘clean’ or ‘empty’ were important factors. Additionally, they indicated that the more realistic illustration of the body on the 3D female body chart (including the perception of skin on the chart) was distracting and unpleasant. For those who believed that the best reproduction of their pain pattern was on the 3D female body chart, the contours, and location of muscles and joints enabled a more personal and accurate communication of their pain. In line with this preference, the simplicity, the lines, and the lack of detail in the 2D female body chart increased the difficulty for communication of their pain. The suggestions for improvements were access to zoomed (enlarged) images of specific body parts and visibility of structures under the skin, such as the muscles, tendons, and bones.

**Table 3:** Part I: Five emergent themes from the semistructured interviews regarding male and female body charts (left column).

Qualitative data from semistructured interviews			
Themes	Examples^a^		
	Comparative/ambivalent	Feminine body chart	Masculine body chart
Differences between body charts	Comparative: *That is a man and that is a woman* *I don’t think that there is a big difference between the two body schemas* *I only noticed the difference afterwards* [when the body schemas were presented simultaneously]	*She has hips and breasts* *She has feminine curves* *That looks like a ladies buttocks*	*He has no hips* *He has no waist* *He is like a square, a box* *The lines on the man seemed like they were out of place*
Preference (explained)	Ambivalent: *It’s a random choice* *It doesn’t matter – I have pain no matter which one I choose* *I didn’t notice that it was a man and a woman*	*This feels right because it’s a woman…it’s more natural for me* *This one reminds me of myself. It looks more like a woman* *It is of 100% importance that it’s a woman* *I’m a woman. That’s why I choose the woman*	*That’s the one* [body schema] *you usually get* *The man is more anonymous than the woman and that’s why it’s easier to draw on the man*
Identification with body charts	Ambivalent: *It’s not important to identify with the body schema* *It’s just a figure* *I can identify with both body schema*	*I can better identify with the female body schema because I’m a woman* *It’s more personal and feminine* *It makes more sense*	*I can see myself in him…when I see myself from the outside then I see myself as bigger – that’s why it’s easier to reproduce the pain and explain the pain*
Drawing experience/accuracy		*It was easier to draw on the woman because I have pain in my hips…* [the hips] *are missing on the man* *I can find the exact spot where the pain is* *I can add more details* *It’s difficult* [to draw] *on the woman because she is more real – I can better locate where my pain is, and I know what that feels like on my own body*	*The man is easier to draw on because he is larger…It’s important that there is a lot of space* [to express the pain] *It’s strange to draw on a man* *It required more thought* [to draw on the man] *I was aware that my drawing on the man didn’t turn out the way I wanted…he was wrong and I couldn’t draw the way I wanted* *It describes something that doesn’t really exist when it’s drawn on a man*
Improvements	Indicating left and right on the body chart (confusion) Better marking of the spine/skeleton Show front and back on the same screen Possibility for more colors for different pain qualities/intensities A larger/thicker pen Adding hair on the head (suggestion from a cancer survivor)		

^a^Quotes from patients are displayed within each theme and divided into responses/opinions to the feminine and masculine body charts as well as comparative/ambivalent responses.

**Table 4:** Part II: Five emergent themes from the semistructured interviews regarding 2D and 3D body charts (left column).

Qualitative data from semistructured interviews			
Themes	Examples^a^		
	Comparative/ambivalent	2D^b^ female body chart	3D^c^ female body chart
Differences between body charts	Comparative: *3D looks more realistic whereas 2D looks artificial* *3D is a figure with skin…it’s alive…2D is more flat* *Maybe 3D is more personal and 2D is less personal* *Not much of a difference – they have the same shape*	*More anonymous* *Appears more clinical* *More tangible* *More like a sketch* *More natural* *Contrasting colors* *Looks like a man* *Just a drawing*	*A real person* *More human* *More detailed* *More serious* *Looks more realistic* *It has calming colors* *It looks too much like a human or a man* *It looks grey…alien-like* *Looks like a robot woman*
Preference (explained)	Ambivalent: *they’re both OK to draw on* *It doesn’t matter which one I draw on*	*I have seen this one more often so it’s natural for me* [to draw on] *It’s just more clear* *I just don’t like the other one*	*It looks more realistic* *It gives a better overview* *You can relate to it because it has skin* *It’s just a little prettier*
Identification with body charts	Ambivalent: *No, I didn’t think about if I could identify with the body schema* *There is no difference in identification with either body schema* *Relating to the body schema is not important*	*I can see myself in this one* *I can better relate to the structure* *The body schema can be anyone – It’s more anonymous* *It’s like it’s not a person* *You don’t really sense that it’s your body*	*It’s like me…It looks like a human being* *I can see myself…I wish that I looked like that* *I can identify with the 3D figure – that’s what makes the difference* *It just seems totally wrong because it’s not me*
Drawing experience/accuracy		I *t’s easier to see where it hurts. It’s more detailed inside* [the body] – *my pain is inside* *It’s easier to draw on the 2D - absolutely…The lines help to specify the location* *It’s easier to explain and draw the pain on the 2D* *It’s easier to draw on the 2D because there is nothing on it* *It’s too much like the VAS scale* *The lines* [on the abdomen] *are annoying* *It’s harder to see where I should draw* *I can’t see what the pain looks like*	*I feel like I draw more and more pain areas – I become more focused* *It’s a more accurate reproduction* [of the pain] *It’s easier to draw on the 3D…and to make others understand where the pain is* *Here it’s easier to see where the muscles are compared to the 2D* *I feel like the pain is more present on the 3D* [I can see] *the elbows, see the shapes, and sense the shoulder blades* *I only see skin on the 3D figure* *It’s unpleasant to draw on another person* *She looks real – that’s distracting*
Improvements	Using an image of one self Split the figure into sections (arm/leg/torso/head) Possibility to “take the skin off” to show muscles and bones (the pain is on the inside) Another skin color on the 3D (it’s too pale) Zoom of different areas to enable a more detailed pain drawing Show side view of the body Possibility for more colors for different pain qualities/intensities Indicate the depth of the pain		

^a^Quotes from patients are displayed within each theme and divided into responses/opinions to the female 2D and 3D body charts as well as comparative/ambivalent responses.

^b^Abb: two-dimensional.

^c^Abb: three-dimensional.

## Discussion

### Preference Tendencies for Gender-Specific Three-Dimensional Body Charts

This study investigated the differences and similarities in drawing behavior, preference, and perception of masculine versus feminine and 2D versus 3D female body charts. Drawing behavior, assessed by reliability analysis, showed very high consistency between masculine and feminine body charts, though pain areas were drawn slightly larger on the feminine body chart, and this error (bias) became gradually larger as the pain area increased in size. When asked about preference, 6 participants preferred the masculine and 35 preferred the feminine body chart. However, the semistructured interviews revealed that only 2 participants truly preferred the masculine, 18 preferred the feminine, and 21 did not really have a preference. Drawing behavior between 2D and 3D female body charts showed very high consistency, though pain areas were drawn marginally larger on the 3D female body chart. When asked about preference, 18 participants preferred the 2D and 23 preferred the 3D female body chart. However, the semistructured interviews revealed that 16 participants truly preferred the 2D, 20 preferred the 3D, and 5 did not really have a preference. The analysis of the semistructured interviews revealed five emergent themes for both masculine versus feminine and 2D versus 3D (Differences between body charts, Preference (explained), Identification with body charts, Drawing experience/accuracy, and Improvements). In summary, the analysis of the quantitative and qualitative data showed that it was important to have a female body chart for women to express their pain correctly; however, with regards to the 2D and the 3D versions of the body chart the preferences were high for both body charts and were driven by factors dependent on the pain depth or location.

### Transition From Masculine to Feminine Body Chart

The majority of research uses a masculine or androgynous body chart to quantify pain areas [8-11], which may prevent women from providing an accurate representation of their pain areas. Expressing pain can be very difficult and verbal language is often insufficient to capture the full pain experience [19,20]. Women with chronic pain communicate the nature of their pain experience with more emphasis on the affective dimension of pain [17,21,22] where men focus on the sensory dimension [22]. As a consequence, chronic pain patients are often misunderstood by health care professionals and have a desperate need to express and explain their pain in hopes of a diagnosis or to establish a symptom management plan [23,24]. Although the consistency between the masculine and feminine body charts was high, the results from the qualitative analysis showed that when women were drawing on the masculine body chart it required more thought to project their pain and the masculine body chart limited detailed expressions. Drawing on the feminine body chart, however, was more natural and ‘makes more sense’. Additionally, the error in the drawn areas between the masculine and feminine body charts became larger as pain areas increased in size, which supports the notion that the use of gender-specific body charts matters. In fact, the results support that women may actually underestimate the extent of their pain distribution on masculine body charts. However, this may also be an indication of that widespread or multisite pain is more difficult to reproduce on 2 consecutive pain drawings than focal pain [14]. Whether this could increase the likelihood that women’s pain drawings are labelled as ‘nonorganic’, a condition that has been suggested to stem from a psychosocial disturbance, given that the criterion for this condition is a wide distribution of pain over many anatomic regions [25], is unclear. In this study, the reported difficulties for women drawing on male body charts were expressed as a lack of female anatomy and identification with the body chart. This raises the possibility that masculine body charts may distort the shape or pattern of the pain areas as they are drawn and moreover, how they are ultimately perceived. A logical next step would be to explore site-specific driven investigations within multiple homogeneous groups. For example, is the need for masculine and feminine body charts equally relevant when reporting knee pain versus hip/groin pain?

Half of the participants indicated that use of the feminine body chart was important; however, a couple of participants preferred the masculine body chart due to familiarity and anonymity. The need for anonymity creates a distance to the pain, which may serve as a coping mechanism and be related to women’s desire to conceal their pain from others [21]. Given that nearly half of the participants had a clear preference for the feminine body chart, with the remaining having no preference, the appropriate choice would be to have gender-specific body charts. Further, when assessing the reliability of the masculine and feminine body charts, the fixed bias was small (˂1% pixel density) and the variance in drawing size is similar, which indicates that the transition from a masculine to a feminine body chart would not introduce significant distortions in the data. On the contrary, the advantages of using gender-specific body charts may facilitate communication and enhance clinical insight. A limitation of this study, however, was that participants were not offered an androgynous version of a body chart when selecting a preference, and thus those that were indifferent may have preferred an androgynous version.

### Transition From Two- to Three-Dimensional Female Body Chart

Three-dimensional image technology has the advantage of adding more detail and realistic representations of the body. Body charts using different 3D techniques, such as contoured sketches [26], photographs [27], and 3D illustrations or body charts [28-35] have been developed, and are generally preferred by patients [31,35]. This study showed that the preference for 2D versus 3D body charts was dichotomous, meaning that 50% of participants preferred the 3D and 40% preferred the 2D (10% did not have a preference). Both body charts portrayed female anatomy, hence the preference appears related to implicit attitudes and personal perception of the body charts and which one (2D or 3D) allowed for the most accurate representation of their pain. Further, it was evident from the qualitative data that identification with the body chart was important for most participants. Drawing one’s pain on a body chart requires an imagined spatial transformation of the body, so-called self-other transformations. When performing self-other transformations different reference frames can be employed. An allocentric reference frame codes object-to-object relationships where both an observers and one’s own perspective is used; whereas an egocentric reference frame codes relationships according to the body axes on the self. Women are more likely to use an egocentric reference frame [36], which could be interpreted as identifying with the body chart, and may enable them to express the affective dimension of the pain experience more easily [17,21,22]. When identifying with the body chart, participants expressed that it was easy to draw an accurate representation of their pain pattern but when identification was not felt, the drawing experience was reported to be ‘wrong’ and ‘unpleasant’. It would be of interest to further discern which types of patients, as defined by their symptoms/diagnosis, render the body charts less effective for communicating pain, why this is so, and which solutions can be developed to overcome the lack of identification.

The 2D and 3D body charts are the same size, the difference being the contours on the 3D body chart. When assessing the reliability the fixed bias was small (˂1% pixel density) and they produce similar variance, which would suggest that the 2D and 3D body charts can be used interchangeably. However, given the dichotomy of the preference for 2D and 3D body charts and that the drawing experience can be affected by the choice of body chart, both body charts should be presented to participants and the appropriate choice should be based on preference. An overall technical limitation of this study is that the location and shape of the pain area itself were not systematically compared. If indeed the 3D body charts provide more guidance for the patients then shape or distribution around, for example, the knee joint or lower back it may impart new meaning and significance.

### Perspectives for Gender-Specific Pain Drawings

Medical treatment has shifted from being a person-oriented qualitative approach where the patient was perceived as a person to an object-oriented quantitative approach where the patient is perceived as a case [37]. The focus of objectivity in medical practice has nearly excluded the patient’s voice in medical knowledge [37]. However, the subjective pain experience cannot be assessed by current medical technology or imaging, rendering it “invisible” to clinicians, hence the diagnosis of musculoskeletal disorders relies largely on the patient’s narrative. If the patient's narrative is not heard fully or understood, the possibility of diagnostic and therapeutic error increases [38]. In the same manner as medical imaging, pain drawings can be used to observe and compare differences over time, both for the clinician and the patient and possibly be a tool to align expectations to treatment outcomes.

It is generally accepted that pain and unpleasantness, sensory disturbances, or symptoms are difficult to verbally express [24], and often patients feel that their vocabulary does not fully capture the pain experience [19,39]. Visual communication thus appears as a means to overcome the verbal language barriers and facilitate an understanding of pain and illness [39]. Integrating the use of pain drawings into primary care, and not just in secondary care where it is used more often, may provide health care professionals with useful information, which assists in the clinical reasoning about the origin(s) and cause of pain, ultimately leading to an early diagnosis and appropriate pain management strategy.

### Conclusion

The main quantitative difference between the masculine and feminine body charts emerged when patients reported larger areas of pain. The qualitative findings of this study further support the need for gender-specific body charts as a tool to facilitate communication of pain. Given the dichotomy of preference, 2D and 3D body charts should be used according to the individual’s preferences. Detailed and accurate pain drawings may lead to improvements in pain communication, and thus facilitate clinical reasoning and treatment strategies. In addition, providing gender-specific body charts will allow participants the opportunity to identify with the body chart and enhance their ability to communicate their pain.

## Abbreviations

2D: two dimensional
3D: three dimensional
ICC: intraclass correlation coefficient
LOA: limits of agreement
SD: standard deviation
